# Stepwise Management of a Rare Case of Unilateral Temporomandibular Joint Ankylosis in an Adult After a Road Traffic Accident: Experience at a Bahrain Government Hospital

**DOI:** 10.7759/cureus.116750

**Published:** 2026-09-22

**Authors:** Nadia Sikri, Zainab Naser, Eman Abdulredha Hasan, Abhishek Ghosh

**Affiliations:** 1 Oral and Maxillofacial Surgery, Salmaniya Medical Complex, Manama, BHR

**Keywords:** interpositional gap arthroplasty, intracapsular condylar fracture, restricted mouth opening, temporalis myofascial flap, temporomandibular joint ankylosis

## Abstract

Intracapsular condylar fractures (ICF) can progress to temporomandibular joint ankylosis (TMJA). Although rare, TMJA is a serious complication that can cause restricted mouth opening, impaired mastication, compromised oral hygiene, and reduced quality of life. Here we report a case and its step-wise surgical management, wherein a 28-year-old male patient suffering from joint ankylosis following a road traffic accident was treated with gap arthroplasty with a temporalis myofascial flap. At one-year follow-up, the patient had a 42 mm maximal interincisal mouth opening with no associated pain on jaw movement. The patient had persistent deviation of the mandible to the left on mouth opening. There was maximal intercuspation upon occlusion, as was before surgery. Mastication was subjectively evaluated with the help of the patient. He reported no difficulty in biting and chewing food normally and had made no changes in his diet post-surgery. At the one-year follow-up, all the ipsilateral facial nerve branches demonstrated normal function.

## Introduction

Condylar fractures account for approximately one-third of mandibular fracture presentations, either isolated or with symphysis-parasymphysis fractures [1]. Intracapsular condylar fractures (ICFs) comprise approximately 65% of these condylar fractures [2]. Management options for ICFs include closed reduction with early mobilization or open reduction with internal fixation. Although management remains controversial, closed treatment is commonly used for intracapsular fractures, with treatment individualised according to the fracture pattern and functional findings [3,4]. Regardless of the treatment method chosen, one of the most serious complications of ICFs is temporomandibular joint ankylosis (TMJA) [5]. This report describes the management of unilateral post- traumatic TMJA following ICF sustained in a road traffic accident. The patient was initially managed at an outside center and referred to our tertiary center upon development of ankylosis of the joint. We have further detailed the current departmental protocol for the management of ICFs, supported by available literature.

## Case presentation

A 28-year-old man presented to the oral and maxillofacial surgical department after referral from the primary health care center in June 2025. He had been involved in a road traffic accident in December 2024. At that time, the patient reported bleeding from his left ear. The bleeding, upon assessment by an ENT surgeon, was reported to be from the anterior wall of the external auditory meatus. There was no abnormality detected in the tympanic membrane after thorough examination. The CT scan ruled out any temporal bone or skull base injuries. He was advised to use ear drops and follow up at the center after four weeks. The CT scan revealed a left condylar fracture. The patient reportedly had restricted mouth opening due to pain but satisfactory occlusion. No surgical intervention was performed, and the patient was discharged with oral antibiotics and analgesics as he had multiple skin abrasions and soft tissue contusions from the accident. He presented upon referral to our center with complaints of progressively decreasing mouth opening. 

Upon examination, the patient had stable occlusion, good oral hygiene, restricted mouth opening (12 mm interincisal), and mild midline deviation of the jaw towards the left side on opening of the mouth (Figure 1).

**Figure 1:**
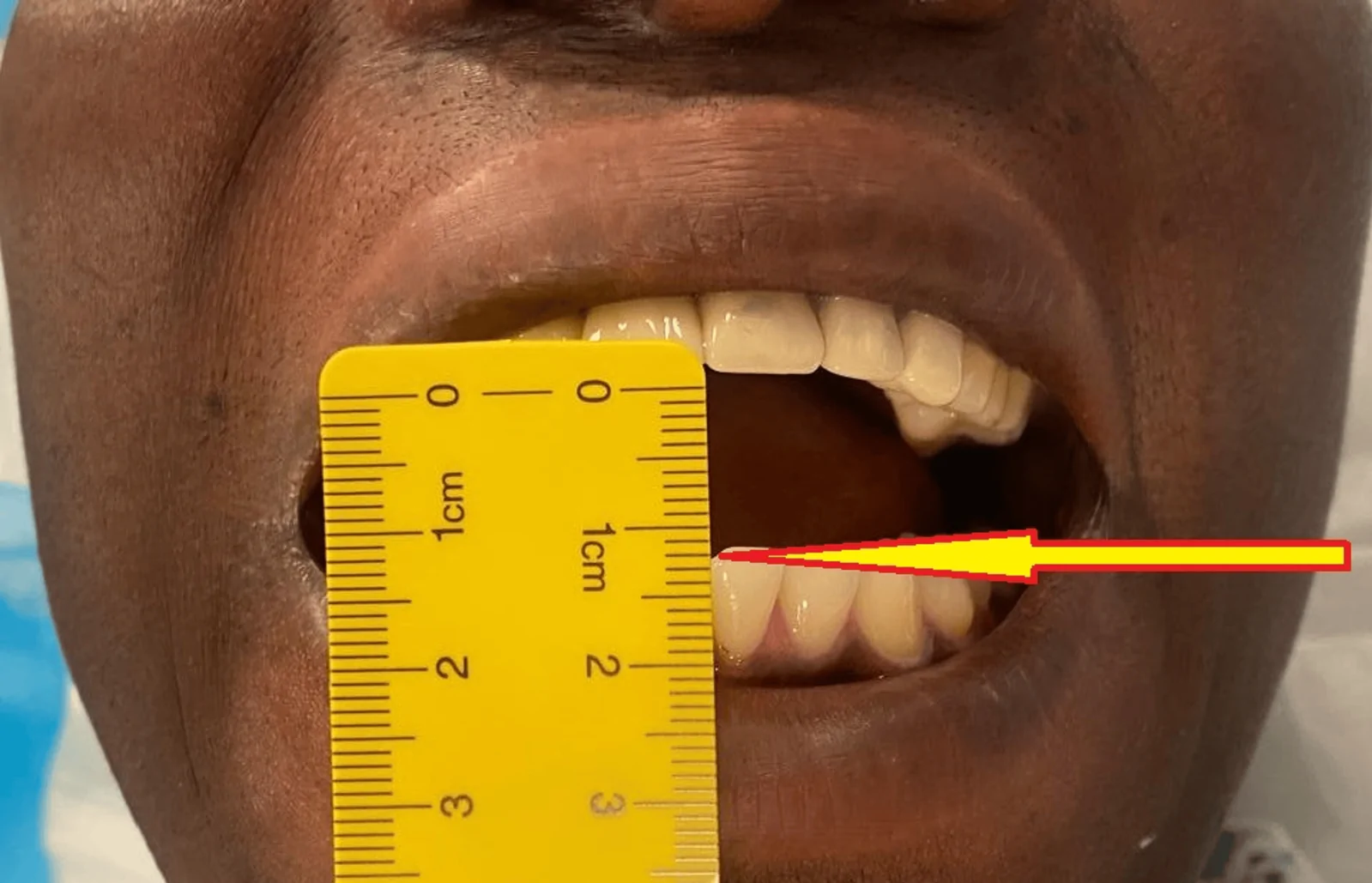
12 mm preoperative mouth opening

There was no palpable joint movement on the left side with opening and closing of the mouth. Orthopantomogram (OPG) and CT facial bone revealed left-sided TMJA (Figures 2, 3). The coronal section of the CT scan was suggestive of a laterally displaced ramal stump in contact with the glenoid fossa via newly formed bone, and a medially displaced, fractured segment of the condyle. The new bone formation extended latero-medially from the lateral-most point on the zygomatic process of the temporal bone to approximately 3.5 cm medially up to the displaced condylar fragment. 

**Figure 2:**
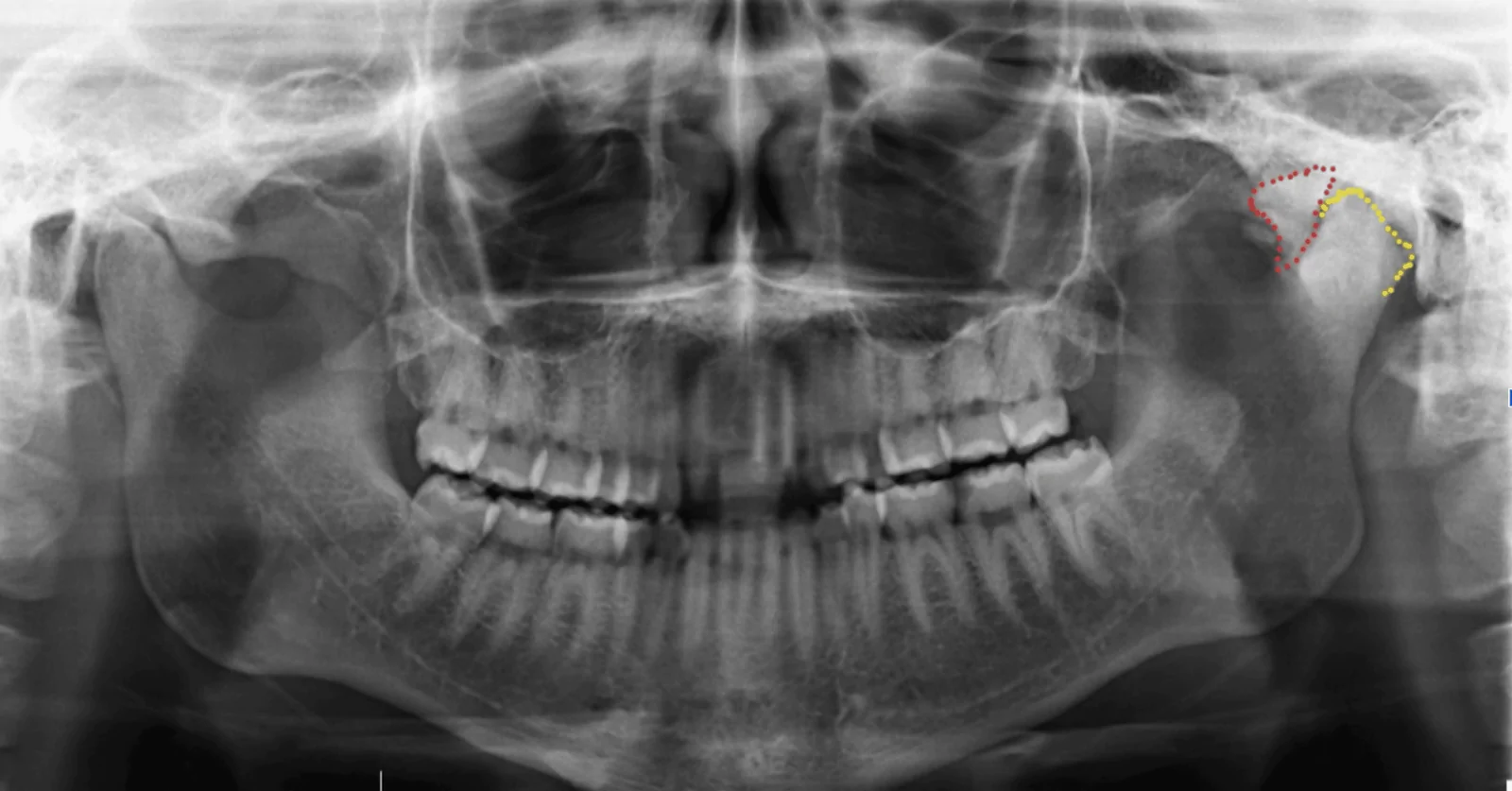
Preoperative orthopantomogram Dotted red line showing a medially displaced fractured medial condylar segment. Dotted yellow line marking the laterally displaced ramal stump coming into approximation with the condylar fossa.

**Figure 3:**
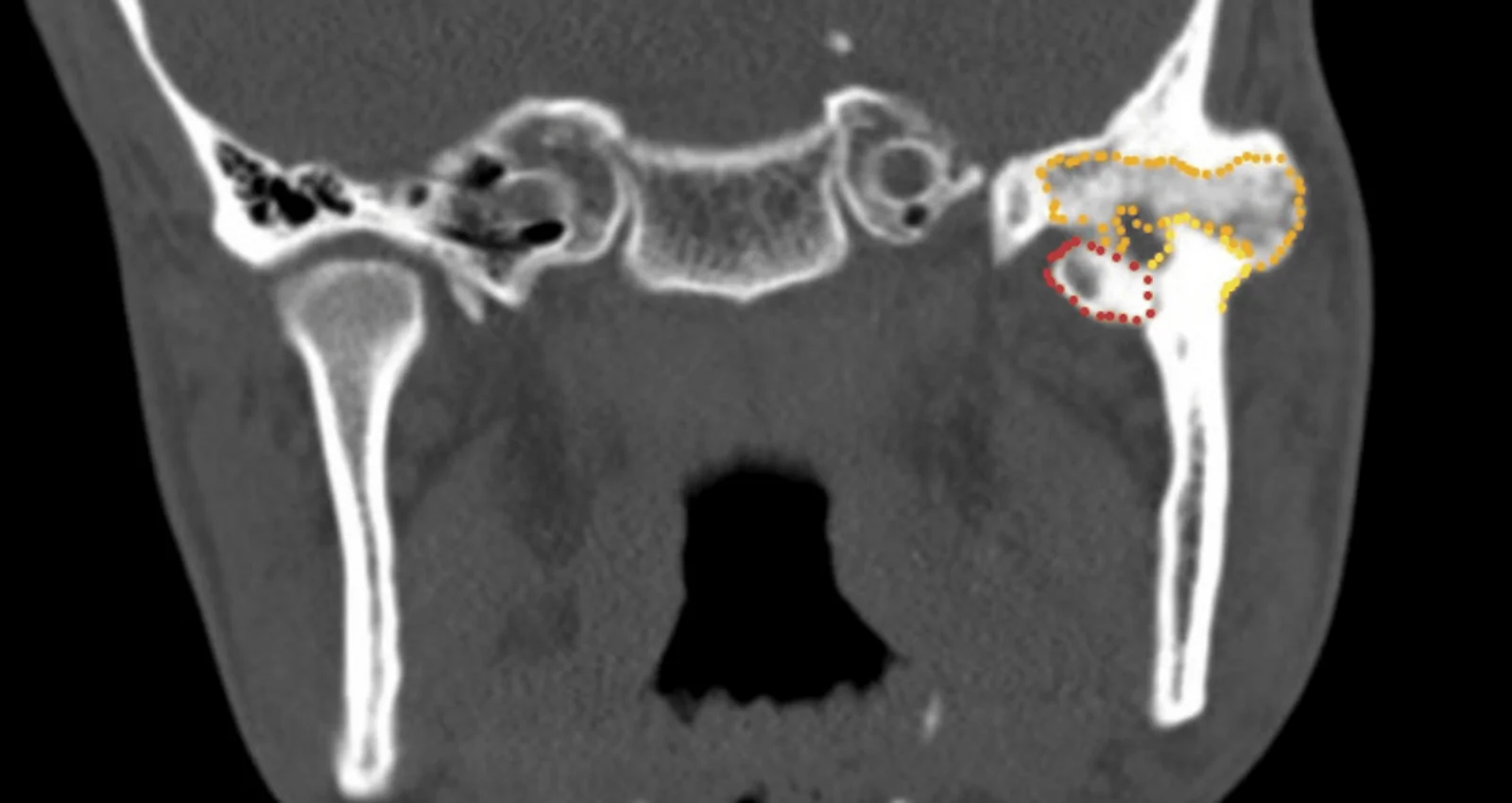
CT scan coronal section Red dotted line marking the medially displaced medial condylar fractured segment. Yellow dotted line marking the lateral ramal stump. Orange dotted line representing the ankylotic mass- new pathological bone formation.

The patient was informed of the same, and a decision was taken to perform ankylosis release surgery with temporomandibular joint gap arthroplasty with an interpositional temporalis myofascial flap, and surgical consent was obtained for the same. All the possible complications were discussed in detail with the patient, including but not limited to facial nerve branch injury and recurrence of ankylosis.

The patient was taken to the operation theater. General anesthesia was induced via nasal intubation with the help of a flexible fiber-optic scope. Alkayat-Bramley modified pre-auricular incision was marked along with other anatomic landmarks. The question mark-shaped skin incision started superiorly approximately pinna’s length from the auricle, supero-anteriorly within the hairline, and the posterior curve was designed to avoid the branches of the superficial temporal vessels. The incision then curved down to the preauricular skin crease (some surgeons prefer endaural extension) (Figure 4).

**Figure 4:**
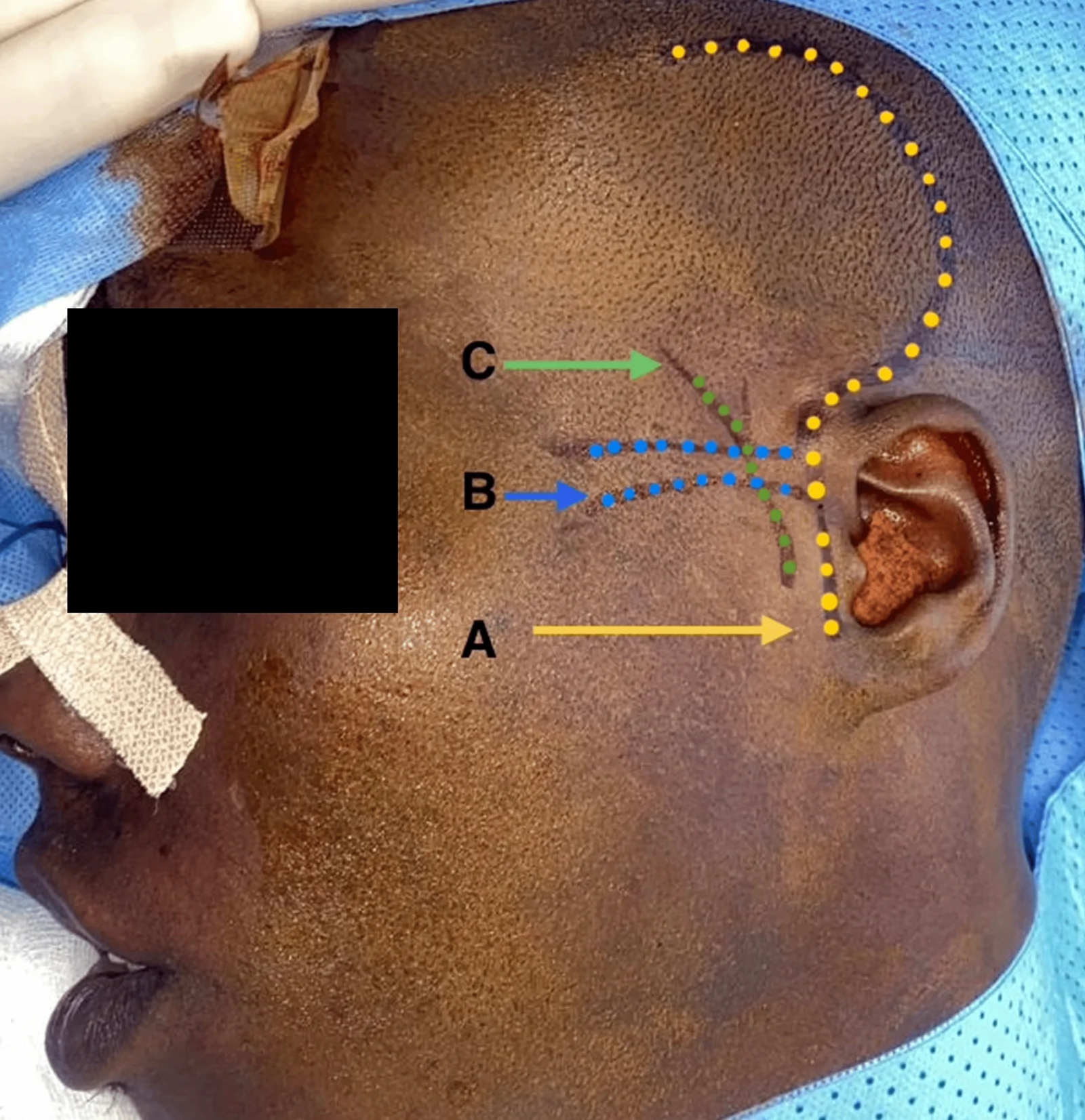
Marked skin incision along with palpated anatomical landmarks (Betadine-soaked gauze piece was placed in the external auditory meatus to prevent blood from entering the canal and clotting) A. Alkayat-Bramley incision with pre-auricular extension inferiorly B. Palpated temporal process of zygomatic arch C. Anterior branch of superficial temporal artery.

The incision was deepened, and dissection carried in the plane between the temporoparietal fascia and the glistening white temporalis fascia. The very thin temporoparietal fascia, or the superficial temporal fascia, is very easy to miss; however, the entirety of this fascia was included with the flap, as the temporal vessels run between this fascia and the subcutaneous fat and the facial nerve branches run deep to the fascia. The blunt dissection was stopped approximately two centimeters above the malar arch (Figure 5).

**Figure 5:**
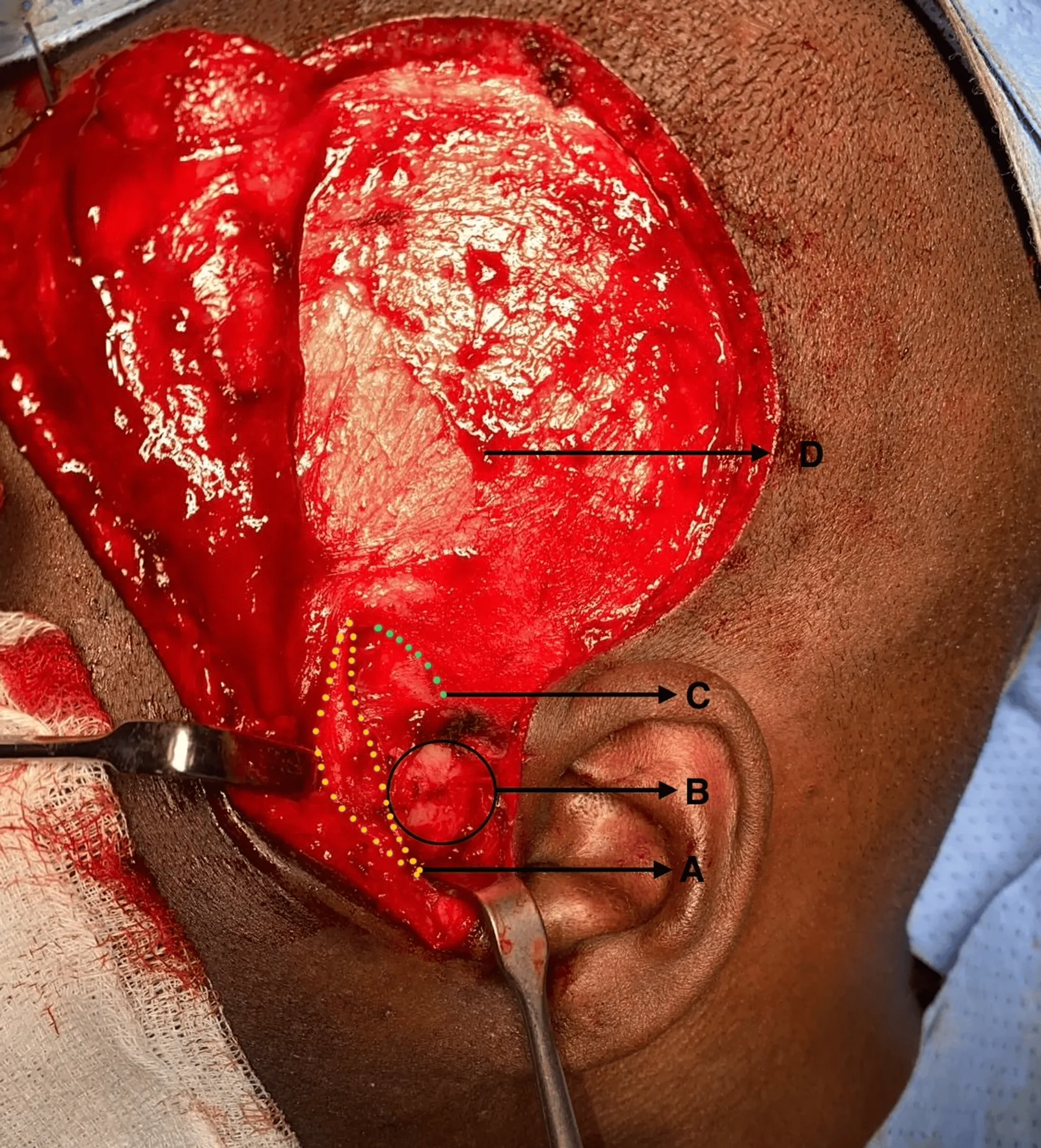
Intraoperative photograph showing the planes of dissection A. The superior layer of temporalis fascia was incised and retracted, marked by the yellow dotted line. B. The ankylotic mass exposed. C. A 45-degree incision was placed from the root of the zygomatic arch on the superior layer of temporalis fascia to carry out blunt dissection in the sub-fascial layer to expose the ankylotic mass. D. The temporalis muscle and glistening white fascia.

Anatomically, the temporalis fascia splits here into superficial and deep as it attaches to the superior aspect of the zygomatic arch, forming a pocket filled with fat. From the root of the arch, an incision at an angle of 45 degrees was placed through the superficial layer of the temporalis fascia, and the fat pocket was breached (Figure 5). From here on, the inferior dissection was carried out in this layer, exposing the ankylotic mass. The fascial incision was extended posteriorly and inferiorly with a vertical arm of approximately 1 cm paralleling the skin incision to facilitate the dissection. However, it was made sure that the incision didn’t cross the attachment level of the earlobe inferiorly. 

The fascial flap was retracted, exposing the ankylotic mass. Upon exposure, a block of the mass measuring approximately 3.5 cm in depth (measured from the zygomatic arch, which is the most lateral point) was carefully drilled out with the sigmoid notch as the anterior landmark and posteriorly protecting the cartilaginous part of the external auditory meatus with retractors. While drilling the bone mass as we neared the medial end, we placed a soft-tissue retractor medially (deep) to protect against accidental injury to the branches of the maxillary artery.

After complete resection, the interincisal mouth opening was checked, which had improved only to 26 mm. Then, an on-table decision was taken to do ipsilateral coronoidectomy as per Kaban’s protocol, which states that ipsilateral coronoidectomy has to be considered if the unforced maximal interincisal mouth opening after resection of the ankylotic mass is less than 35 mm (Figure 6A, 6B) [6]. 

**Figure 6:**
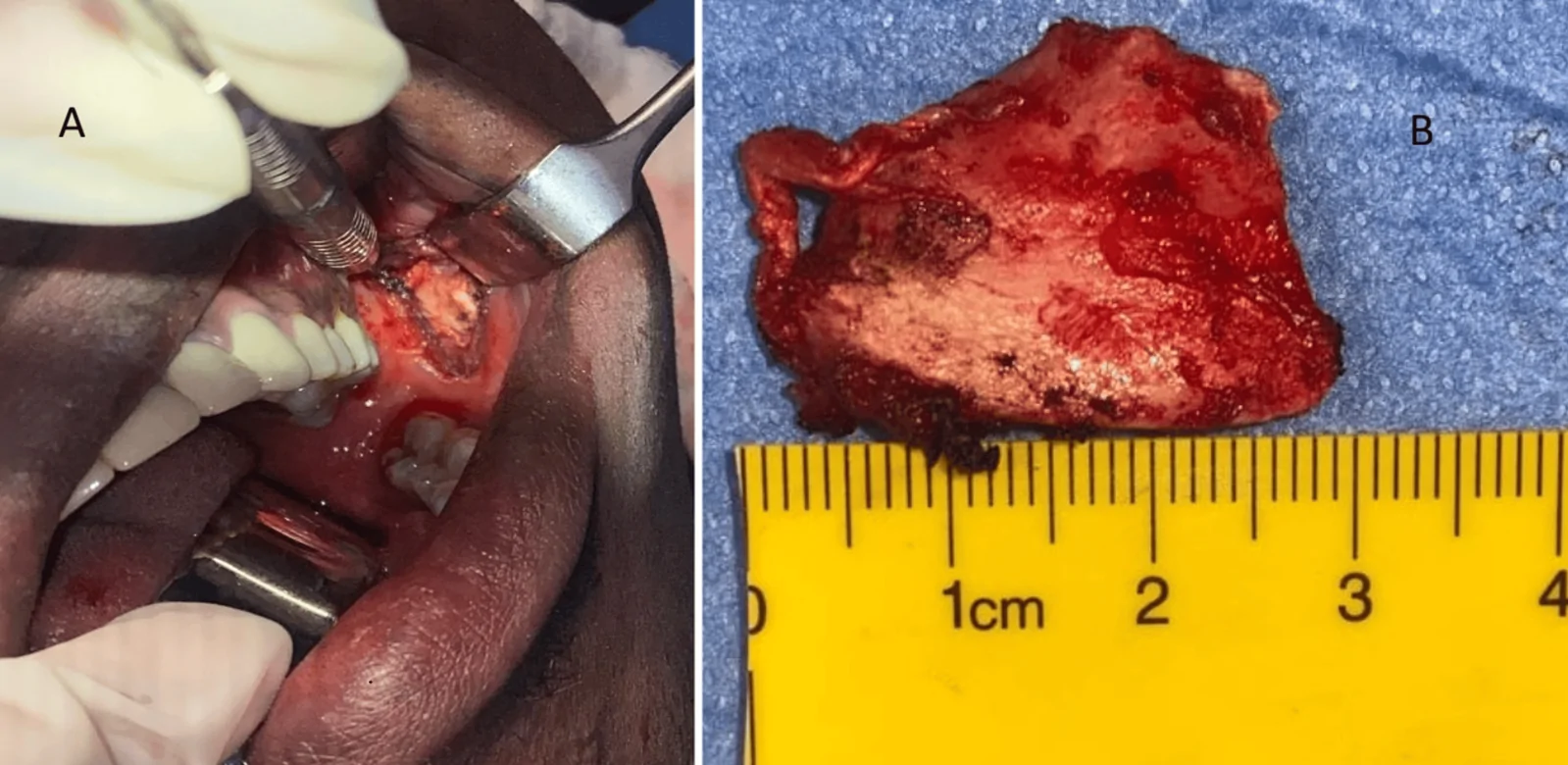
Intraoral approach for coronoidectomy A. Intra-oral approach: incision over anterior border of ramus in open mouth position. B. Sectioned coronoid.

After ipsilateral coronoidectomy, the mouth opening improved to 40 mm interincisal (Figure 7). 

**Figure 7:**
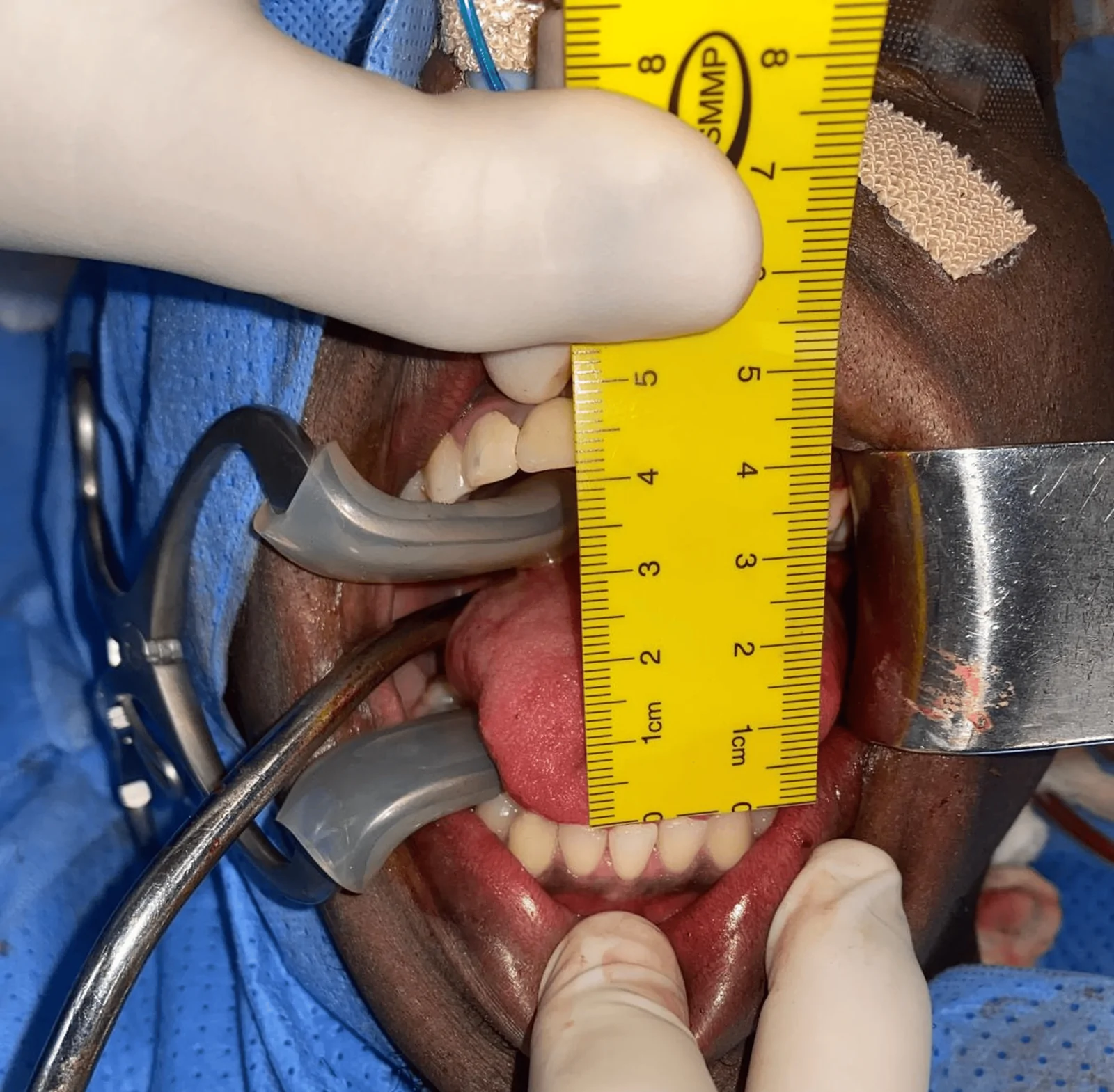
Intraoperative mouth opening post coronoidectomy

Then a thorough wash was given at the surgical site, and the intra-oral wound was closed. Hemostasis was achieved in the joint area, and a temporalis myofascial flap was harvested and inserted into the gap created and stabilised with sutures. While harvesting the flap, care was taken not to accidentally take part of the cranial periosteum, as it has osteogenic potential (Figure 8).

**Figure 8:**
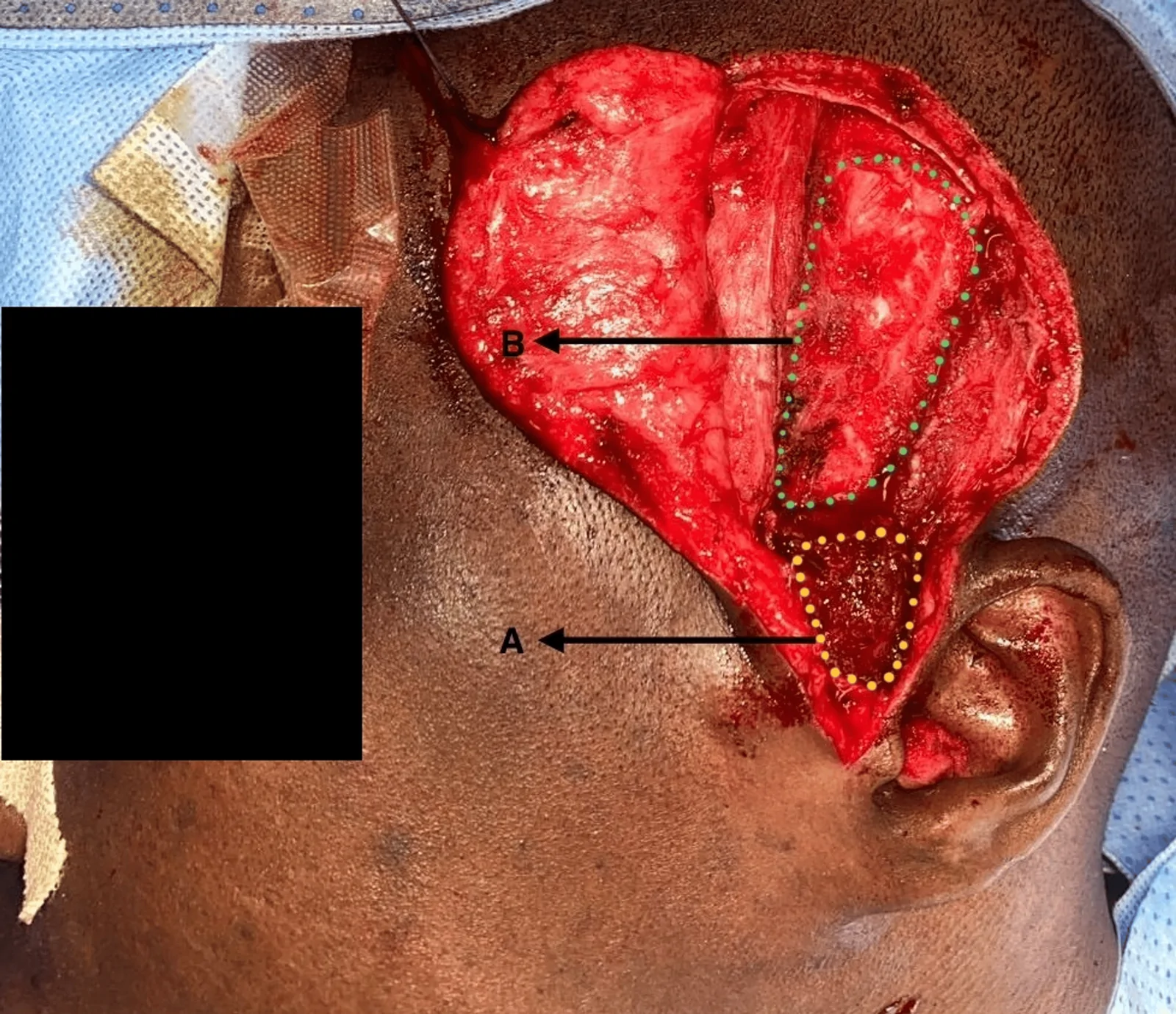
Temporalis myofascial flap harvested A. Temporalis myofascial flap folded over the temporal process of the zygomatic arch and inserted into the gap. B. Cranial periosteum seen as bed after harvesting temporalis myofascial flap.

The wound was closed in layers over the suction drain (Figure 9A, 9B).

**Figure 9:**
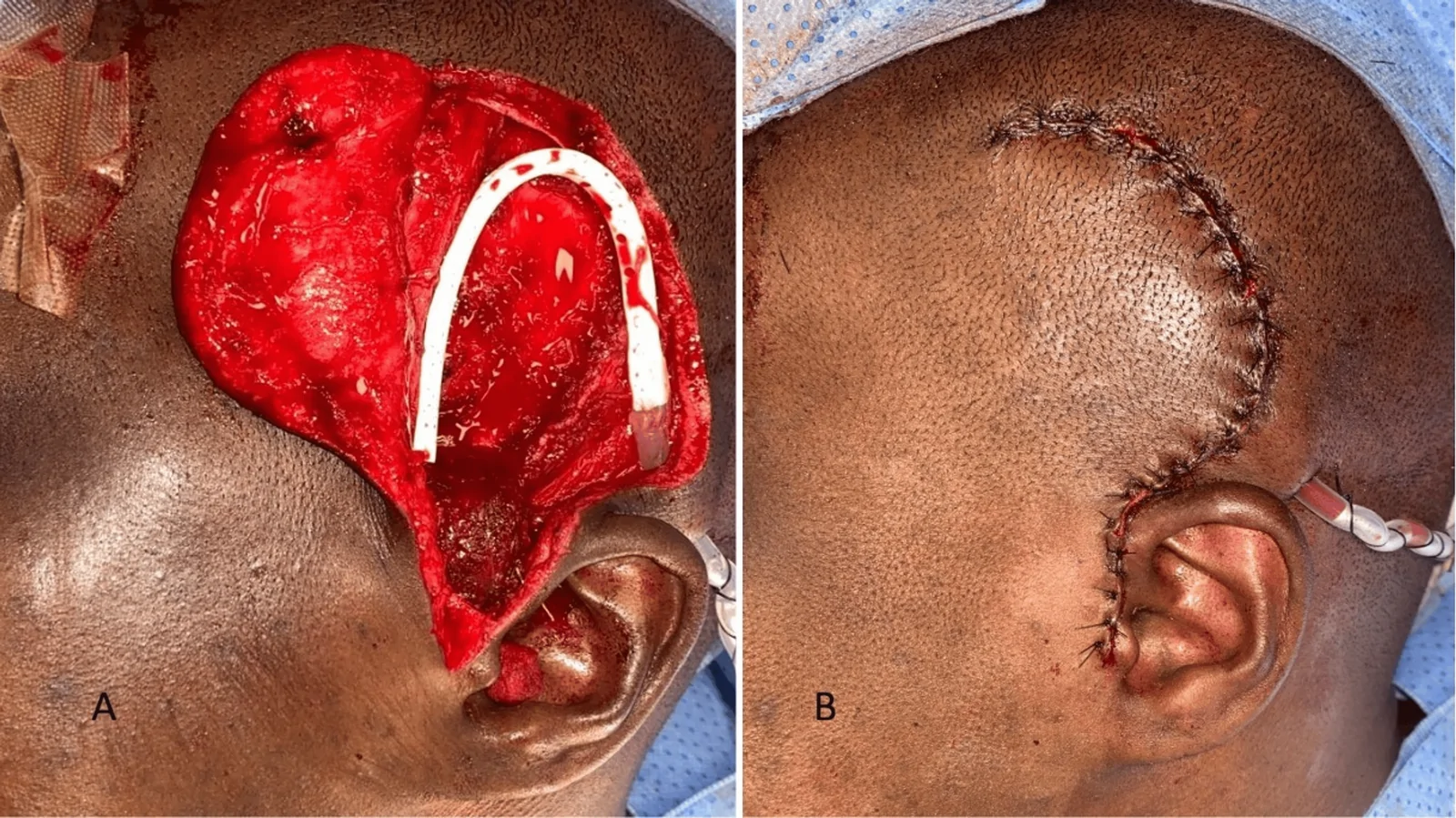
Placement of drain and wound closure A. Drain placed B. Final closure

The patient was put on rigorous physiotherapy from postoperative day 2, wherein he started a soft diet and jaw-opening exercises. The exercises included active excursions, hinge openings, and manual stretching with placement of a stacked bundle of wooden tongue spatulas in front of a mirror in increasing numbers till the intended interincisal opening was achieved, four to five times daily for up to 10 minutes (Figure 10A). During the next month, the diet was changed to the patient's regular presurgical diet. The patient was advised to do hot fomentation over the surgical site after the exercise sessions for muscle relaxation. The patient was also encouraged to do gum chewing exercises. The patient was followed up every month for six months and then after one year. At the one-year follow-up, the patient was encouraged to continue with the exercises on his own for at least two sessions daily of 10 minutes each. The functioning of the facial nerve branches (temporal and orbital) was also recorded and found to be normal (Figure 11A, 11B). The patient had a maximal inter-incisal mouth opening of 31 mm at one month post-op and 42 mm at one year post-op (Figure 10A, 10B). He had no associated pain on jaw movement. There was persistent deviation of the mandible to the left on mouth opening. Maximal intercuspation was observed upon occlusion, as was before surgery. Mastication was subjectively evaluated with the help of the patient. He reported no difficulty in biting and chewing food normally and had made no changes in his diet post-surgery. At the one-year follow-up, all the ipsilateral facial nerve branches demonstrated normal function.

**Figure 10:**
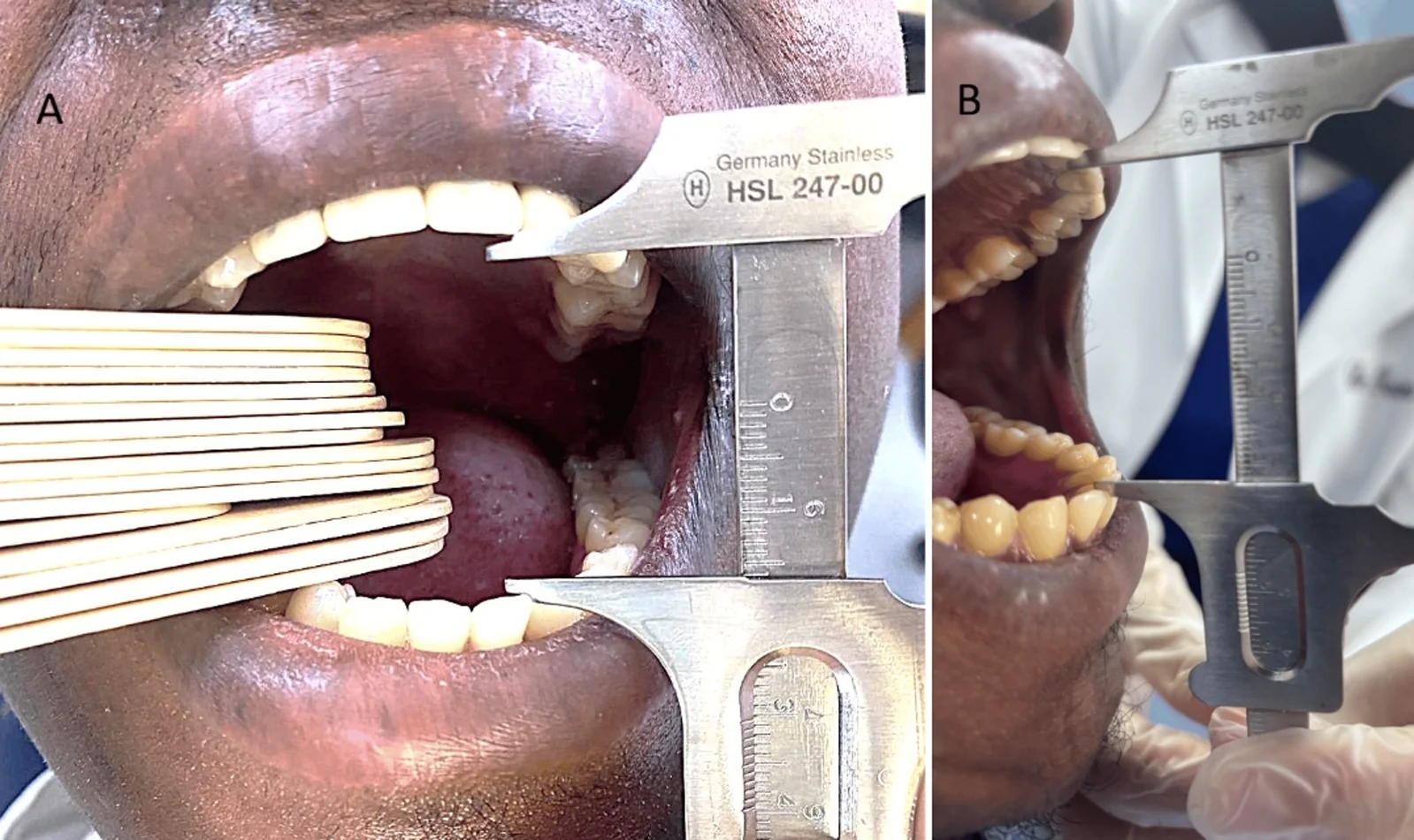
Post-operative inter-incisal mouth opening A. 31 mm interincisal mouth opening at one month post-op B. 42 mm interincisal mouth opening at one year post-op

**Figure 11:**
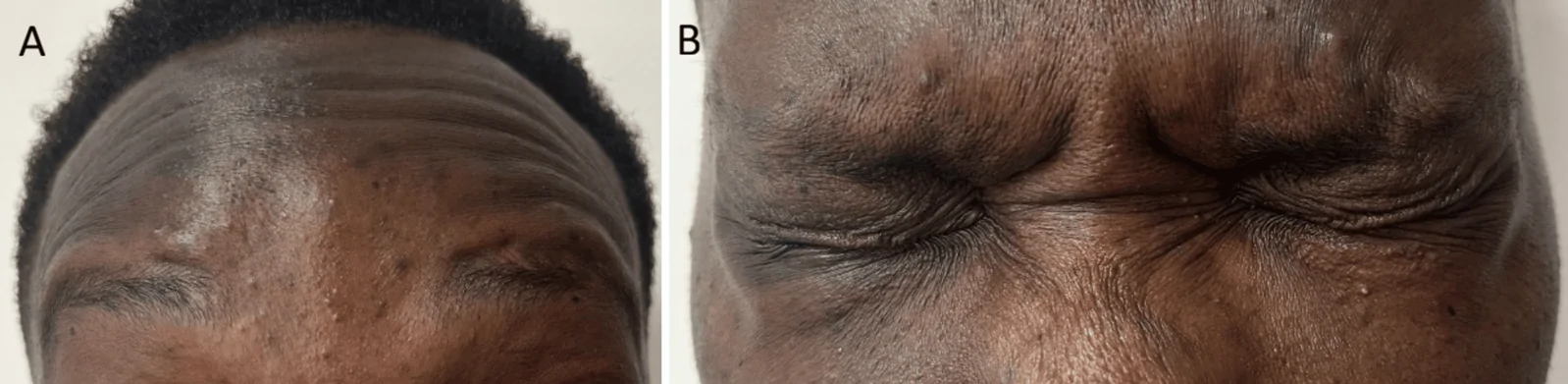
Facial nerve branches’ function A. Normal function of temporal branch of facial nerve depicted B. Normal function of orbital branch of facial nerve depicted

## Discussion

One of the most common craniofacial disorders is ankylosis of the temporomandibular joint. Development of TMJAs is insidious, leading to non-diagnosis by both clinicians and patients during the early stages until it presents as proper restricted mouth opening [7]. The rate of TMJA following condylar fracture is < 5%, but it is a serious complication as it affects nutrition, speech, esthetics, and maintenance of oral hygiene [2,8-10]. Although there is no conclusive evidence of one factor leading to ankylosis after condylar fractures, multiple factors have been identified as important. Age is the most cited one; in children, the condyle is highly vascularised with a tremendous ability to form new bone. Secondarily, damage to the joint architecture, the glenoid fossa, condylar cartilage, and perforation or displacement of the articular disc. The third factor is prolonged mechanical immobilization of the joint [2]. 

Currently, an orthopantomogram in conjunction with a CT scan is used as a standard in radiological evaluation of condylar head fractures as a prerequisite to their subsequent management [11]. Condylar head fractures have now been essentially recognised as “shear fractures” with sagittal forces acting in a cranio-lateral to medio-caudal direction [11]. Spiessel and Schroll in their classification (1972) have classified the condylar head fracture as type (VI). It was later modified by Neff and Rasse (2006), wherein type (VI) was further categorized into type (VI)A - displacement of the medial condylar pole with preservation of the vertical dimension and type (VI)B - lateral condylar pole is involved with loss of the vertical dimension [11]. Our case post facto was classified as type (VI) B. Dongmei et al. further classified the relation of the stump of the ramus to the glenoid fossa, where Grade 0 = no contact between the stump of the ramus and the fossa, although the stump is in the fossa; Grade I = contact is there between the stump and the fossa, and the stump is still in the fossa; and Grade II = stump of the ramus is laterally displaced out of the fossa [2]. Interestingly, Dongmei et al. proposed a classification that can further guide our treatment approach. They divided the condylar head into thirds (lateral, middle, and medial). Then they classified ICFs into three types. Type A fracture line passes through the lateral third and results in reduction in ramal height. Type B fracture line passes through the middle third without reduction in ramal height. Type C fracture passes through the medial third without reduction in the ramal height. They further suggest open reduction and internal fixation for type A fractures; however, they advise closed reduction for type B and C fractures, as reducing and stabilising a medially displaced fractured segment is technically very challenging [2]. The absolute indication for open reduction, as per the authors, is lateral displacement of the ramal stump and its approximation to the glenoid fossa that makes the probability of TMJA very high [2].

When we analysed the pre-operative coronal CT scan of our patient, we identified a medially displaced condylar fragment, suggestive of a medial pole condylar fracture, along with ankylotic mass formation between the laterally placed ramal stump and the glenoid fossa. Our case was a type C fracture but had some reduction in ramal height, as suggested by mild midline deviation of the jaw towards the left side. Thus, according to us, there is no universal classification that can ideally characterize all types of ICFs. Larger multi-center studies need to be conducted to develop a universal classification. Every patient needs to be evaluated on a case-by-case basis to decide the treatment approach. However, as Dongmei et al. suggested, the absolute indication for open reduction will be the approximation of the laterally displaced ramal stump to the glenoid fossa [2]. Open reduction of ICFs can be challenging in type B and type C fractures, wherein the fractured condylar segment has displaced medially and anteriorly. In such cases, achieving an ideal reduction with stabilisation can prove very challenging with conventional temporomandibular joint surgical approaches. Thus, the authors agree with Dongmei et al., wherein such cases are treated with closed reduction and early mobilisation.

There is no one-size-fits-all approach when dealing with these fractures, and it needs to be custom-tailored according to age/level of compliance and overall health of the patient. Prolonged inter-maxillary fixation (IMF) should be avoided even at the cost of deranged occlusion [12,13].

## Conclusions

TMJAs are a rare complication of ICFs. In our institute, for unilateral ICFs, a maximum period of 10-14 days of IMF is done, followed by guiding elastics if the occlusion is deranged, and aggressive physiotherapy. The guiding elastics are changed every three days on an outpatient basis as per institutional protocol.

The physiotherapy department constantly upgrades its standard operating protocols as per international standards in the rehabilitation exercises for the TMJA patients. The patients post-operatively are put on a close follow-up schedule and regularly reinforced about the importance of mouth opening exercises as a part of the home oral care regimen. This is our anecdotal institutional protocol and needs to be further validated by larger multi-institutional studies. With the above case report, the authors intend to guide the upcoming trainee maxillofacial surgeons in the complex surgery of TMJA in a stepwise manner to facilitate its easy execution and to cultivate interest in this rare and complex pathology.
